# A Prospective Study Evaluating Gender Differences of Serious Outcomes through Difficult Airway Physiological Score (DAPS) in the Emergency Department

**DOI:** 10.1155/2024/4622511

**Published:** 2024-05-20

**Authors:** Shahan Waheed, Rida Jawed, Ahmed Raheem, Asad Iqbal Mian

**Affiliations:** Department of Emergency Medicine, Aga Khan University and Hospital (AKUH), Karachi, Pakistan

## Abstract

**Introduction:**

Gender variation in critically ill adults after resuscitation is reported in many studies. However, this variation is not well established when evaluating the physiological instability in this population. This study aimed to prospectively evaluate the gender variation in serious outcomes by the difficult airway physiological score (DAPS) among critically ill patients requiring endotracheal intubation (ETI).

**Methods:**

This is a cohort study conducted from August 2021 to December 2022 in the emergency department of Aga Khan University. The prospective validity of the difficult airway physiological score was derived using retrospective data and includes 12 variables: sex, age, time of intubation, hypotension, respiratory distress, vomiting, shock index >0.9, pH < 7.3, fever, anticipated decline, Glasgow Coma Scale (GCS) < 15, and agitation. The serious outcomes were cardiac arrest, mortality (within 1 hour after intubation in emergency), hypotension (systolic blood pressure <90 mmHg), and oxygen desaturation (SpO_2_ < 92%). The difference between males and females was assessed using the chi-square test, and the association of gender and serious outcomes was explored using Cox and logistic regression analysis. ROC curve analysis and area under the curve assessed score validity separately in males and females with serious outcomes.

**Results:**

We enrolled 326 patients with a mean age of 50.3 (±17.8), with 123 (33.7%) females and 203 (62.2%) males. 198 (60.7%) patients were >45 years old, of which 136 (67%) were male and 62 (50.4%) female. Cardiac arrest was observed in 56 (17.2%), with 24 (19.5%) females and 32 (15.8%) males, *p* value 0.348. Hypotension after intubation was observed in 132 (40.5%) patients, 56 (45.5%) females and 76 (37.4%) males, *p* value 0.149. Oxygen saturation (<92%) was observed in 80 (24.5%) patients, 32 (26%) females and 48 (23.6%) males, *p* value 0.630. In females, the DAPS of 11 had an area under the curve of 0.863 (0.74–0.91). The sensitivity of the score was 84.8%, the specificity was 71.9%, the PPV was 77.8%, and the NPV was 80.4% with an accuracy of 78.9%. In males, the DAPS score of 14 had an area under the curve of 0.892 (0.57–0.75). The sensitivity of the score was 67%, the specificity 93.8%, the PPV 92.2%, and the NPV 72.2% with an accuracy of 79.8%.

**Conclusions:**

The Difficult Airway Physiological Score (DAPS) predicts the risk of serious outcomes after intubation with high precision and reliability with different score cutoffs between the two sexes, highlighting the gender variation of a difficult airway.

## 1. Introduction

Airway management is a critical component of basic resuscitation and is crucial to patient safety and optimum clinical outcomes [1–3]. Establishing and maintaining a patent airway to ensure adequate oxygenation and ventilation is the first step of emergency medicine care [4]. However, the encounter with a difficult airway during induction or emergence poses significant challenges and requires a systematic approach for first-pass success [5]. The timely identification of a challenging airway can allow emergency medicine physicians to anticipate potential difficulties and adopt appropriate strategies to mitigate risks and improve patient safety [6]. Airway behavior in humans is influenced by both biological (sex-related) and sociocultural (gender-related) determinants throughout their lifespan [7]. Understanding these relationships is essential for interpreting gender-based anatomical variations and in studying their association with the occurrence of a difficult airway [7, 8].

Traditionally, anatomical airway classifications, such as the Mallampati score [9] or thyromental distance [10, 11], have been employed to predict difficult airways. However, these scoring systems often fail to account for differences that may occur specifically in male or female patients. Recent studies have suggested that gender-related factors could play a crucial role in determining the likelihood of a difficult airway, prompting the need for a comprehensive investigation of these disparities [12, 13]. The Difficult Airway Physiological Score (DAPS) is a useful tool designed to assess and predict the difficulty of airway management before intubation. The DAPS score was derived using retrospective data and includes 12 variables: sex, age, time of intubation, hypotension, respiratory distress, vomiting, shock index ≥0.9, pH < 7.3, fever, anticipated decline, Glasgow Coma Scale (GCS) < 15, and agitation. By assigning points to each parameter, DAPS allows clinicians to objectively assess the potential challenges they may face and to make informed decisions regarding the choice of airway management techniques and devices, ultimately ensuring better intervention and treatment of patients.

This study aims to explore and compare the determinants of difficult airway between male and female patients using the DAPS scoring system. By focusing on gender-based disparities, the study seeks to fill the current knowledge gap and provide information on how various factors of the DAPS score may predict difficulties in airway management in a gender-specific way.

## 2. Methods

### 2.1. Study Design and Setting

A prospective cohort validation of DAPS was conducted in the emergency department (ED) of Aga Khan University Hospital from August 2021 to December 2022. The recruiting center is an urban, academic, 62-bed emergency department that receives 60,000 patients annually. The inclusion criteria of our study were all adult patients (≥18 years) who came to the ED and required endotracheal intubation (ETI). Patients with oropharyngeal tumors that require advanced airway measures due to distorted anatomy, patients with a history of cardiac arrest outside the hospital with ongoing CPR, and pregnant females due to varied physiological derangements were excluded from the study. The intubation criteria were severe respiratory distress, worsening hypoxia that did not respond to noninvasive positive pressure ventilation, GCS less than 8, anticipated decline (intubation based on physician discretion), and imminent airway compromise. We estimated our sample size to be 268 based on an absolute precision of 6% with a 95% confidence interval and a 5% level of significance. The sample size was calculated from a study by Smischney et al. [14] by the WHO calculator, showing a 52% rate of postintubation hypotension. The initial calculation of the sample size relied on specific assumptions, including expected effect size, variability, and anticipated dropout rates. However, during the study, a higher-than-expected enrollment rate of patients and lower dropout rates emerged, and the choice to enroll a greater number of patients than originally calculated was made to strengthen the robustness and applicability of our results. This decision was motivated by the increased statistical power derived from a larger sample, facilitating a more comprehensive exploration of gender differences in serious outcomes using the DAPS in the emergency department.

The Ethics Review Committee of Aga Khan University approved the study (ERC Number 2020-4975-14778). Consent was taken from the patient if he or she has intact capacity or from the accompanying attendant, who is the patient's decision-maker in the emergency department visit.

### 2.2. Data Collection

The triage nurses and the resuscitation room doctors identified patients who needed intubation in the emergency department and informed the researchers. The associates screened the patients after obtaining verbal consent from the patient (with intact capacity, which was understanding, appreciation, reasoning, and expression of choice about the process followed in the study) or the accompanying decision-maker, which was later followed by a written consent from either. Preintubation vitals at the triage were recorded followed by other demographic variables. During the collection of variables, the research associates did not interfere with the treatment of patients requiring ETI. Data were collected on a pretested questionnaire that was tested in data collection to derive the physiological score of difficult airways. The data collected on the form were reviewed by the physician involved in the ETI to review missing data and confirm the data. Symptoms and vital signs at presentation, reason for intubation, difficult airway evaluation, drugs used in ETI, and other procedural data were collected. The information collected was periodically reviewed by the principal investigator for accuracy. The patient was followed in the emergency department 15 minutes and 1 hour after intubation for record of vitals, and the final disposition was recorded on an electronic medical record. The estimated risk of serious outcomes associated with each level of score in the derivation study was not in the data collection form to prevent physicians from making treatment decisions based on the risk score.

### 2.3. Serious Outcomes

The primary outcomes were worsening hypotension and hypoxia. Hypotension was defined as a decrease in the systolic blood pressure (<90 mmHg), and hypoxia was defined as peripheral oxygen desaturation (<92%) within one hour of intubation. Secondary outcomes were cardiac arrest (defined as the absence of pulse after ETI in a critically ill patient in the ED) and mortality (defined as death occurring within 1 hour after intubation). All of the above outcomes were measured at different times: immediately after intubation, and 15 minutes and 1 hour after intubation.

### 2.4. Statistical Analysis

The study utilized Redcap for data entry and SPSS-22, along with Python 3.8.14, for analysis. Descriptive statistics were used for continuous and categorical variables, with comparisons made using appropriate tests (chi-square, Fisher's exact, *t*-test, or Mann–Whitney *U* test). The association between gender and serious outcomes was explored using binary logistic regression models. Variables known or suspected to be associated with serious outcomes were examined with univariate binary logistic regression. Independent variables with a *p* value of <0.05 univariate regression were included in the multivariate model. Multivariate models were constructed using stepwise backward selection. Only variables with a *p* value of <0.05 were retained in the final model. The goodness of fit was measured using the Hosmer–Lemeshow test, which evaluates the agreement between observed and predicted outcomes. The Nagelkerke *R* square and overall correct classification percentage were also reported for each model. The difficult airway physiological score was assessed using the ROC curve analysis, determining the AUC with 95% confidence intervals. Youden's J statistic identified the main discriminating point of the DAP score, and sensitivity, specificity, PPV, and NPV were calculated at various cutoff points, all with 95% confidence intervals. A significant level of 0.05 was applied.

## 3. Results

In this study, 326 patients were enrolled, of which 123 were women and 203 were men. The average age of the patients was 50.3 years, and the women had a slightly lower average age of 45.6 years compared to the men with an average age of 53.1 years.

The most common reasons for intubation were shortness of breath in 239 patients (73.3%) and then coma in 220 patients (67.5%), followed by respiratory distress in 165 patients (50.6%), anticipated decline in 143 patients (43.9%), hypoxia in 130 patients (39.9%), and metabolic acidosis in 89 patients (27.3%), followed by trauma with no significant differences between the sexes. Most of the patients had a shock index below 0.9 (54.3% of the patients).

Table 1 presents the baseline characteristics of critically ill patients who required ETI. In general, the baseline characteristics of the critically ill patient, such as preintubation vitals, pH levels before intubation, and the HEAVEN criteria did not show significant differences between sexes, except for age.

Table 2 shows the gender variation in the serious outcomes of the patients who required ETI with cardiac arrest, postintubation hypotension (SBP < 90 mmHg), and low oxygen saturation (<92%) with no significant statistical differences between sexes.

Table 3 shows the univariate and multivariate logistic regression analysis to predict serious outcomes after ETI in men with shift duty (from morning to night from 8 am to 10 pm), shortness of breath, fever, drowsiness, trauma, others (unspecified), hypoxia, anticipated decline, respiratory distress with significant association of serious outcomes in both univariate and multivariate logistic regression analysis, with isolated trauma, pH group <7.3, shock index >0.9, hypoxemia, extreme size, cardiac arrest, hypotension (SBP < 90 mmHg), and oxygen saturation (<92%) showing association only in univariate logistic regression analysis.

Table 4 shows univariate and multivariate logistic regression analysis to predict serious outcomes after ETI in women <45 years of age, with shift duty (from morning to night) from 8 AM to 10 PM, shortness of breath, fever, drowsiness, seizures, trauma, coma, altered mental status (GCS < 15), metabolic acidosis, respiratory distress, pH group < 7.3, and shock index > 0.9 were significantly associated with serious outcomes after ETI.

**Figure 1:**
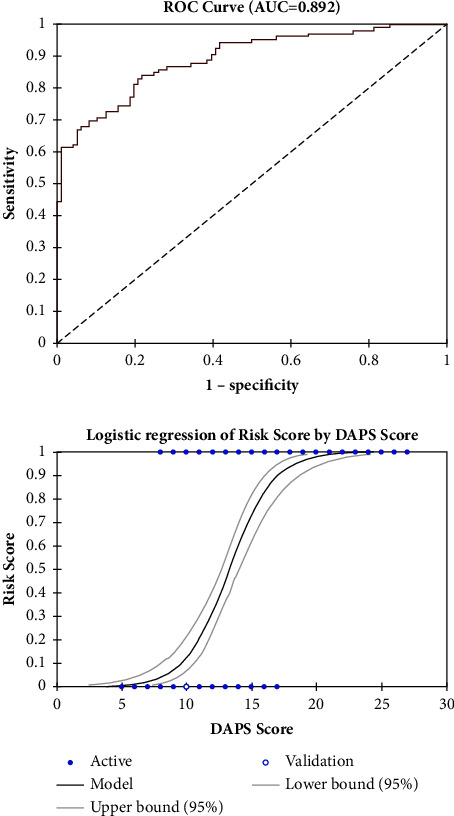
Receiver operating characteristic (ROC) curves for the prediction of serious outcomes among men after ETI in the ED with an area under the curve of 0.892.

**Figure 2:**
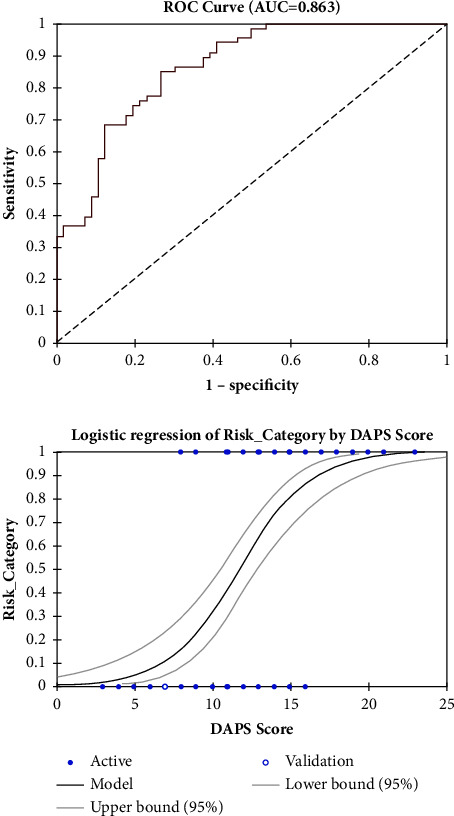
Receiver operating characteristic (ROC) curves for the prediction of serious outcomes among women after ETI in the ED with an area under the curve of 0.863.

The area under the curve (AUC) of 0.892 in Figure 1 and the AUC of 0.863 in Figure 2 suggest that the predictive model of the DAPS score used in this study has high precision in distinguishing male and female patients. The sensitivity of the DAPS score of 11 in women was 84.8%, specificity 71.9%, PPV 77.8%, and NPV 80.4% with an accuracy of 78.9%, while the sensitivity of the DAPS score of 14 in men was 67%, specificity 93.8%, PPV 92.2%, and NPV 72.2% with an accuracy of 79.8%.

## 4. Discussion

The Difficult Airway Physiological Score (DAPS) exhibits excellent accuracy, with a high AUC, in predicting which patients are likely to experience severe consequences after ETI, with a specific score differentiation between male and female patients. The score has been developed and validated for difficult intubation in the emergency department, as a simple model that can be easily applied in clinical practice. A DAPS score of 11 in women has an accuracy of 78.9%, a sensitivity of 84.8%, a specificity of 71.9%, a PPV of 77.8%, and an NPV of 80.4%, while a DAPS score of 14 in men has an accuracy of 79.8%, a sensitivity of 67%, a specificity of 93.8%, a PPV of 92.2%, and an NPV of 72.2%. The study has shown that the DAPS score can predict difficult intubation in the emergency and also reveals a high rate of severe morbidity related to difficult intubation.

Various scores have been suggested to assess the possibility of difficult airways in preoperative and intensive care unit (ICU) settings, like the LEMON score [15], which is the most commonly used tool to assess difficult airways, designed for use in the preoperative clinic setting before elective surgery. Although some of the LEMON criteria (such as the 3-3-2 rule and the Mallampati score) require an awake and cooperative patient, they lack guidance on the anticipated complications that an emergency airway may present.

Our score contrasts with other scores specifically designed for ICU like the MACOCHA score [16], which predicts the difficulty of tracheal intubation in ICU patients. The score is calculated by assigning points to Mallampati class, apnea, cervical spine limitation, mouth opening, coma, hypoxemia, and nonanesthesiologist operator. A higher MACOCHA score indicates a higher risk of difficult intubation. In the primary study [17], the cutoff of three or above rules out difficult intubation, provided a good negative predictive value of 97% and 98% and specificity of 90% and 89%, with sensitivity of 76% and 73% but low positive predictive values of 48% and 36% in the original and validation cohorts, respectively. In addition, there is no gender-based anatomical and physiological scoring, which, as our data suggest, has a significant impact on the parameters and outcome of intubations.

Furthermore, our data compared with the HYpotension Prediction Score (HYPS) [18] that predicts hypotension before and after intubation in the ICU setting, determining a total of 11 adverse hypotension factors after intubation, namely, increased age, APACHE II score, sepsis, intubation performed in settings of cardiac arrest or MAP 65 mmHg or decreasing SBP from 130 mmHg, acute respiratory failure, diuretics 24 hours before ETI, catecholamines or phenylephrine 60 minutes before ETI, and etomidate as sedative. The score has a PPV of 11.9% and an NPV of 88.1% in the lowest category, and a PPV of 71.9% and an NPV of 28.1% at the highest risk threshold [14]. However, this only serves as a caution to one of the many adverse outcomes of difficult intubation and does not account for gender differences either.

On the other hand, the HEAVEN criteria [19] (hypoxemia, extremes of size, anatomical challenges, vomit/blood/fluid, exsanguination/anemia, and neck mobility problems) was the first tool, with data collected from emergency medical services (EMS) patients, offering a more practical and useful tool for emergency airway management than other tools that were developed in a more controlled setting. Multiple studies [20–22] have been done to test the clinical significance of the criteria that found that the physiological factors of hypoxemia and exsanguinations are not associated with failure of first-attempt intubation. The Difficult Airway Physiological Score (DAPS) expands on the parameters of different identifiers of the difficult airway, resulting in a more comprehensive and superior predictability score.

The prediction of airway compromise is more imperative in an emergency setting than in a nonemergency setting. The DAPS score provides a rapid and reliable prediction of difficult airways in the uncertain, severe, and urgent environment of the ED. It is also a novel score that takes into account several gender-based physiological differences and provides distinct score cutoffs for each gender in the interpretation, reinforcing the disparities between the airway behavior of the two genders.

## 5. Limitations

Although our study provides valuable information on gender differences in serious outcomes related to difficult airway management through the DAPS score, it is important to acknowledge its limitations. As the study has been conducted in a specific geographical area and in a single ED, it limits the generalization of the findings to other settings. The results obtained from a single institution may not be representative of the larger population. Additionally, there may have been a possible selection bias in the study sample. Triage systems are often used in emergency rooms to categorize patients according to the severity of their symptoms. This may result in overrepresentation of certain population characteristics and limit the use of findings in contexts of other settings. Furthermore, the accuracy and reliability of the study scoring system may have been subjected to measurement bias as physiological parameters such as anticipated decline, agitation, and respiratory distress may be interpreted and recorded differently between different medical professionals, resulting in inter-rater variability. Furthermore, in the study, not all possible confounders that could affect the results were taken into account. The analysis could not adequately account for factors such as comorbidities, drugs, prior airway treatments, and the experience of the healthcare provider, all of which may have an impact on outcomes. The ability to conclude the precise impact of gender on adverse outcomes could also have been limited by the lack of a comparison group, such as a control group without challenging airways.

Interpreting the results of our study requires an appreciation of these limitations, and future research should strive to address these issues to provide a more complete understanding of gender differences in difficult airway management outcomes.

## Figures and Tables

**Table 1:** Baseline characteristics of critically ill patients requiring ETI.

Characteristics	Total 326	Gender distribution		*p* value
		Female	Male	
		123	203	
Age (years)	50.3 (±17.8)	45.6. (±18)	53.1 (±17.2)	<0.001^*∗*^
Age groups				
<45 Years	128 [39.3%]	61 [49.6%]	67 [33%]	0.003^*∗*^
≥45 Years	198 [60.7%]	62 [50.4%]	136 [67%]	
Shifts				
Night (10 pm–08 am)	142 [43.6%]	56 [45.5%]	86 [42.4%]	0.577
Morning to evening (08 am–10 pm)	184 [56.4%]	67 [54.5%]	117 [57.6%]	
Preintubation vitals [median (IQR)]				
Systolic blood pressure (mmHg)	128 (150–77)	126 (146.5–82)	131 (151–75)	0.414
Diastolic blood pressure (mmHg)	76 (90–38)	76 (89–36)	75 (90–40)	0.571
Heart rate	110 (125–63)	110 (125–66)	110 (126–60)	0.884
Oxygen saturation (%)	93 (98–57)	93.5 (98–62.5)	93 (98–52)	0.610
Respiratory rate	28 (36–16)	28 (36–18)	28 (35–16)	0.715
Reasons for intubation				
Coma	220 [67.5%]	85 [69.1%]	135 [66.5%]	0.627
Hypoxia	130 [39.9%]	50 [40.7%]	80 [39.4%]	0.824
Metabolic acidosis	89 [27.3%]	37 [30.1%]	52 [25.6%]	0.380
Anticipated decline (deterioration in next few hours)	143 [43.9%]	53 [43.1%]	90 [44.3%]	0.826
Shortness of breath	239 [73.3%]	92 [74.8%]	147 [72.4%]	0.637
Respiratory distress	165 [50.6%]	64 [52%]	101 [49.8%]	0.690
Polytrauma	17 [5.2%]	9 [7.3%]	8 [3.9%]	0.184
Isolated trauma	21 [6.4%]	5 [4.1%]	16 [7.9%]	0.174
Gunshot injury	4 [1.2%]	1 [0.8%]	3 [1.5%]	0.597
Others^*∗*^	8 [2.5%]	2 [1.6%]	6 [3%]	0.452
Shock index				
<0.9	177 [54.3%]	71 [57.7%]	106 [52.2%]	0.333
≥0.9	149 [45.7%]	52 [42.3%]	97 [47.8%]	
pH				
>7.3	74 [22.7%]	26 [21.1%]	48 [23.6%]	0.600
≤7.3	74 [22.7%]	26 [21.1%]	48 [23.6%]	
Heaven criteria				
Hypoxemia	128 [39.3%]	49 [39.8%]	79 [38.9%]	0.869
Extremes of size	27 [8.3%]	11 [8.9%]	16 [7.9%]	0.736
Anatomic abnormalities	46 [14.1%]	16 [13%]	30 [14.8%]	0.656
Vomit/blood/fluid	111 [34%]	46 [37.4%]	65 [32%]	0.321
Exsanguination	12 [3.7%]	5 [4.1%]	7 [3.4%]	0.774
Neck mobility issues	45 [13.8%]	18 [14.6%]	27 [13.3%]	0.735

^*∗*^Worsening type 2 failure, aspiration pneumonia, epileptic status, asthmatic status, severe agitation/diarrhea, impending threat to the airway due to burns or esophageal rupture or expanding hematoma.

**Table 2:** Variation in sex in the serious outcome of patients requiring ETI.

Outcome	Total	Gender		*p* value
		Female	Male	
Cardiac arrest	56 [17.2%]	24 [19.5%]	32 [15.8%]	0.384
Postintubation hypotension (SBP < 90 mmHg)	132 [40.5%]	56 [45.5%]	76 [37.4%]	0.149
Oxygen saturation (<92%)	80 [24.5%]	32 [26%]	48 [23.6%]	0.630

**Table 3:** Univariate and multivariate logistic regression analysis for predicting serious outcomes among men after ETI.

Factors	Univariate		Multivariate—initial level		Multivariate—final level	
	OR [95% CI]	*p* value	OR [95% CI]	*p* value	OR [95% CI]	*p* value
Age ≥45 years	1.54 [0.83–2.86]	0.176				
Shift duty (morning-evening)	4.85 [2.56–9.2]	<0.001^*∗*^	6.85 [2.34–19.99]	<0.001^*∗*^	5.85 [2.2–15.52]	<0.001^*∗*^
Shortness of breath	6.92 [3.43–13.96]	<0.001^*∗*^	1.66 [0.52–5.29]	0.389		
Fever	9.83 [3.38–28.6]	<0.001^*∗*^	14.52 [4.17–50.59]	<0.001^*∗*^	9.78 [3.6–26.57]	<0.001^*∗*^
Drowsiness	2.51 [1.17–5.39]	0.018^*∗*^	0.42 [0.13–1.33]	0.14		
Seizures	0.66 [0.18–2.43]	0.532				
Trauma	0.33 [0.13–0.8]	0.015^*∗*^	0.41 [0.04–4.41]	0.462		
Coma	0.61 [0.27–1.4]	0.245				
Others^*∗*^	2.17 [1.14–4.11]	0.018^*∗*^	0.98 [0.21–1.67]	0.324		
Changed in altered mental status	0.89 [0.47–1.68]	0.723				
Hypoxia	8.72 [3.72–20.45]	<0.001^*∗*^	6.74 [1.79–25.36]	0.005^*∗*^	6.22 [2.27–17.08]	<0.001^*∗*^
Metabolic acidosis	16.94 [3.97–72.25]	<0.001^*∗*^	3.51 [0.61–20.07]	0.159		
Anticipated decline	0.52 [0.28–0.95]	0.032^*∗*^	0.36 [0.13–1]	0.05	0.32 [0.12–0.84]	0.02^*∗*^
Respiratory distress	7.08 [3.59–13.979]	<0.001^*∗*^	3.47 [1.23–9.77]	0.019^*∗*^	2.96 [1.17–7.45]	0.021^*∗*^
Polytrauma	0.43 [0.11–1.793]	0.249				
Isolated trauma	5.71 [1.89–17.235]	0.002^*∗*^	13.73 [1.81––42.29]	0.011^*∗*^	6.97 [1.27–38.28]	0.026^*∗*^
PH group ≤ 7.3	9.47 [2.82–31.851]	<0.001^*∗*^	6.15 [0.93–40.68]	0.059	7.98 [1.66–38.35]	0.009^*∗*^
Shock Index ≥ 0.9	5.1 [2.57–10.09]	<0.001^*∗*^	1.42 [0.48–4.15]	0.525		
Hypoxemia	3.43 [1.71–6.871]	0.001^*∗*^	0.59 [0.17–2.02]	0.404		
Extreme size	0.49 [0.13–1.77]	0.272				
Anatomic abnormalities	0.64 [0.26–1.57]	0.326				
Extinguisher	1.13 [0.21–5.99]	0.886				
Neck mobility issue	0.93 [0.38–2.25]	0.865				
Vomit_blood_fluid	1.65 [0.88–3.07]	0.118				
Cardiac arrest	8.32 [1.92––36]	0.005^*∗*^	5.01 [0.54–46.45]	0.156		
Hypotension (SBP < 100 mmHg)	4.1 [1.97–8.51]	<0.001^*∗*^	1.59 [0.34–7.39]	0.553		
Oxygen saturation (>92%)	6.76 [2.31–19.78]	<0.001^*∗*^	5.01 [0.54–46.45]	0.156		
Death in hospital	1.76 [0.83–3.73]	0.139				
Discharged	0.44 [0.24–0.82]	0.009^*∗*^				

Nagelkerke R square = 0.734, overall correct classification percentage = 86.7%, and Hosmer–Lemeshow test (*p*=0.182). ^*∗*^Worsening type II failure, aspiration pneumonia, status epileptic, status asthmaticus, severe agitation/diarrhea, impending threat to the airway due to burns or esophageal rupture or expanding hematoma.

**Table 4:** Univariate and multivariate logistic regression analysis to predict serious outcomes among women after ETI.

Factors	Univariate		Multivariate—initial level		Multivariate—final level	
	OR [95% CI]	*p* value	OR [95% CI]	*p* value	OR [95% CI]	*p* value
Age ≥45 years	3.87 [1.81–8.3]	0.001^*∗*^	3.65 [0.66–20.28]	0.139		
Shift duty (morning-evening)	5.31 [2.44–11.59]	<0.001^*∗*^	6.07 [1.03–35.87]	0.047^*∗*^	7.85 [1.6–38.38]	0.011^*∗*^
Shortness of breath	7.53 [3.1–18.29]	<0.001^*∗*^	10.79 [1.44–81.05]	0.021^*∗*^	14.25 [2.56–79.33]	0.002^*∗*^
Fever	4.77 [1.8–12.65]	0.002^*∗*^	4.79 [0.79–29.25]	0.09	7.12 [1.63–31.21]	0.009^*∗*^
Drowsiness	5.62 [2.24–14.14]	<0.001^*∗*^	21.62 [2.75–169.94]	0.003^*∗*^	15.57 [3.09–78.36]	0.001^*∗*^
Seizures	0.15 [0.03–0.76]	0.021^*∗*^	0.04 [0–0.54]	0.016^*∗*^	0.03 [0–0.22]	0.001^*∗*^
Trauma	0.23 [0.06–0.93]	0.039^*∗*^	0.04 [0–0.37]	0.005^*∗*^		
Coma	0.34 [0.14–0.86]	0.023^*∗*^	0.33 [0.04–2.4]	0.27	0.04 [0–0.27]	0.001^*∗*^
Others	0.94 [0.46–1.95]	0.871				
Changes in altered mental status	0.64 [0.29–1.42]	0.277				
Hypoxia	1.27 [0.61–2.66]	0.519				
Metabolic acidosis	5.67 [2.15–14.98]	<0.001^*∗*^	4.25 [0.61–29.59]	0.145	6.98 [1.27–38.32]	0.025^*∗*^
Anticipated decline	1 [0.48–2.06]	0.993				
Respiratory distress	5.31 [2.18–12.952]	<0.001^*∗*^	0.99 [0.15–6.47]	0.991		
Polytrauma	0.33 [0.08–1.371]	0.126				
Isolated trauma	0.17 [0.02–1.527]	0.113				
PH group ≤ 7.3	2.89 [1.07–7.808]	0.037^*∗*^	14.15 [0.71–280.72]	0.082		
Shock index ≥ 0.9	4.81 [2.13–10.856]	<0.001^*∗*^	3.63 [0.58–22.81]	0.17	7.02 [1.41–35.12]	0.018^*∗*^
Hypoxemia	0.96 [0.46–1.992]	0.906				
Extreme size	0.79 [0.22–2.86]	0.719				
Anatomic abnormalities	1.11 [0.39–3.22]	0.842				
Extinguisher	1.07 [0.17–6.62]	0.946				
Neck mobility issue	1.5 [0.55–4.09]	0.428				
Vomit_blood_fluid	1.52 [0.72–3.18]	0.269				
Cardiac arrest	2.5 [0.92–6.83]	0.074				
Hypotension (SBP < 100 mmHg)	3.7 [1.71–8.02]	0.001^*∗*^	2.56 [0.52–12.71]	0.25		
Oxygen saturation (>92%)	1.25 [0.55–2.86]	0.597				
Death in hospital	2.24 [1.06–4.7]	0.034^*∗*^	1.64 [0.19–14.38]	0.658		
Discharged	0.55 [0.26–1.17]	0.122				

Nagelkerke R square = 0.809, overall correct classification percentage = 90.2%, Hosmer–Lemeshow test (*p*=0.89, chi-square = 3.615). ^*∗*^Worsening type 2 failure, aspiration pneumonia, status epileptic, status asthmaticus, severe agitation/diarrhea, impending threat to the airway due to burns or esophageal rupture or expanding hematoma.

## Data Availability

All data reported in the manuscript are publicly available in the form of tables and results within the manuscript.
